# Observations in Practice, Surgery, Gynecology, and Especially Obstetrics

**Published:** 1878-03

**Authors:** George B. Walker

**Affiliations:** Professor of Obstetrics in the Medical College of Evansville


### OBSERVATIONS IN PRACTICE, SURGERY, GYNECOLOGY, AND ESPECIALLY OBSTETRICS.

By George B. Walker, M. D.,
PROFESSOR OF OBSTETRICS IN THE MEDICAL COLLEGE OF EVANSVILLE.

(*Read before the Indiana, Illinois and Kentucky Tri-State Medical Society, in Evansville, Oct.* 17, 1877.)

*Continued from page* 176.

### *Descent of Hand and Cord.*—3 *Cases.*

9. The writer was called, June 18th, 1857, to see Mrs. R——, mother of several children, who was in labor. The waters had been drained off three days, the left hand and funis were occupying the vagina, and, as far as could be ascertained from a German midwife, who had been in attendance, had been down for 24 hours. The patient's strength remained good, the pains being strong and almost constant. Although, from the interval since the escape of the water, the case seemed unpromising, version by the feet was resolved upon. The hand being introduced, the right foot was seized and brought down, and the delivery completed in about 15 minutes. The child's hand returned spontaneously to the cavity of the uterus, as the feet came down. It had been dead apparently for 24 hours. The woman made a good recovery.

10. Mrs. P——, aged about 35 years, mother of several children, was taken in labor at 8 a. m., Saturday, January 1st, 1860, and the waters soon came away. The funis and hand were discovered in the vagina, by the midwife in attendance. The writer was called to the case at 2 a. m., on Monday, and proceeded to deliver by version. The right foot was secured and brought down, and the delivery accomplished with reasonable dispatch. The child was still-born, though action of the heart could be discerned for 15 minutes. The uterus contracted promptly, and the woman recovered well.

11. Mrs. G——, mother of three children, was taken in labor on the morning of January 2d, 1873. The right hand and cord descended into the vagina. The patient had taken ergot, and the ergotic pains were well established when the writer was called in, this being at 10 o'clock, a. m. The right shoulder was pressed strongly into the superior strait; no pulsation was perceptible in the cord. The hand being introduced into the uterine cavity, the right foot was grasped and brought down without much difficulty, the occiput coming away in front toward the arch of the pubis. The placenta was soon delivered, and the uterus contracted well. Although the post partum indications were favorable, the woman failed to rally, and died in three days.

*Face Presentation.*—1 *Case.*

12. Mrs. C——, aged 34, had had four children; was taken suddenly in labor, on the night of March 24th, 1858. The membranes broke soon after the accession of labor pains. It was a face presentation, the forehead being posterior and to the right (3d position of the face), the chin anterior, and left of the symphysis pubis. The right foot being secured, was brought down first, and there was nothing untoward in the operation or the result. The woman and child both did well.

*Neck Presentation.*—1 *Case.*

13. Mrs. P——, 23 years of age, had had one abortion and one still-born child. She has had labor pains for the last ten days, sometimes with an interval of half an hour, and sometimes longer; was taken to-day, December 15th, 1875, with strong and frequent labor pains; the os uteri open, and the membranes protruded. The membranes were now ruptured, and the neck and occiput were found presenting at the superior strait, the head lying in the left iliac fossa. After the lapse of half an hour the scalp tumor became large and flabby, sufficiently so to indicate the death of the child; its movements had not been felt by the mother for a week. Considering that the labor had been pretty strong for several days, and the child failing to engage in the strait or promising soon to enter the basin, delivery by turning was supposed to be indicated. The left foot was secured and brought down, and the child taken away without undue trouble. The abdomen of the child was distended with gas, from putrefaction, and was hence its most bulky part, passing through the pelvis with more difficulty than did the head; the skin was loose and readily peeled off; the child was nearly or quite mature. After some unpleasant reverses, probably from toxæmia, the woman at length made a good recovery.

*Inertia.*—2 *Cases.*

14. Mrs. R——, primipara, was seen December 19th, 1857; had been in labor eighteen hours; the child's head had receded from the pelvic brim; os dilated; the waters had been discharged for several hours; the patient was 20 years of age; the child large; the uterine contractions were becoming feeble. The hand was introduced into the uterus, the right foot seized and brought down, and the child delivered. The body came away without trouble, but there was delay in delivering the head. The child died during the operation; the woman's recovery was good.

15. Mrs. R——, aged about 29 years, primipara; was seen in consultation with Dr. Hatchett, at 8 o'clock p. m., March 10th, 1858. She had been in active labor thirty-six hours; the head was engaged in the superior strait, and remained in the same position four or five hours; os uteri moderately open, but rigid; child dead; pains rather strong, but not advancing the child from its position; head presentation, with the occiput anterior and to the left. The hand being introduced into the uterus, the left foot was secured, the turning effected, and the child delivered, the operation requiring about thirty minutes. The child was large, and appeared to have been dead several hours; the woman, having a good constitution, and having, previous to the labor, enjoyed good health, made an excellent recovery.

*Shoulder Presentation.*—3 *Cases.*

16. Mrs. S——, aged 40, mother of several children, has been in labor with moderate pains for several days. This day, December 3d, 1865, the pains have been strong for two hours; gestation of eight months' duration; os uteri moderately dilated and yielding, not without resistance, however; right shoulder presenting, with the head resting in right iliac fossa. The hand was introduced, the right foot secured and brought down, and the delivery effected without difficulty. The child was alive, but small and feeble, otherwise in good condition. On the expulsion of the placenta, a second fœtus came away, apparently at the 6th month; it had probably been dead two months. The two placentas were adherent to each other, but had no vascular union; that of the dead fœtus exhibited fatty degeneration, that of the living child appeared normal.

17. Mrs. T——, aged 38 years, mother of seven children, has been in labor 24 hours, in charge of a midwife at first, afterward of a homeopathic practitioner. The writer was called to see her December 26th, 1872, at 12 o'clock, and found the left side of the head presenting, with the left shoulder pushed strongly into the pelvic basin, the head lying in the left iliac fossa. The patient had taken ergot, and her pains were strong and continuous; strength still good; the cord had been prolapsed three hours; the child was dead. By applying some force, the shoulder was pushed up, and the hand passed through the cervix-uteri; finding the left foot, it was secured and brought down, the blunt hook was used over the right thigh; there was some trouble in getting away the head, but finally the child was extracted, and soon followed by the placenta. The woman's condition after delivery promised a good result, but she sank and died four days afterwards.

18. Mrs. W——, aged about 25, mother of three children, has been in labor about eighteen hours. Dr. Forsmeier had been in attendance. The writer was summoned to visit her at 5 a. m., February 18th, 1873. The right elbow was found presenting at the superior strait, with strong uterine contractions; to verify the diagnosis the child's hand was drawn down. Turning being decided upon, the hand was passed into the uterus, after some resistance from uterine contractions, the right foot seized and brought down, and the child delivered. The child was dead, the cord being destitute of pulsation at the commencement of the operation; the woman recovered without further trouble.

*Contracted Pelvis.—4 Cases.*

19. Mrs. R——, German, aged about 25 years, first child; the sacro-pubic diameter of the brim was contracted. The writer was called to see her on Tuesday afternoon, April 28th, 1856. The woman had been in labor forty-eight hours, the first twentyfour in charge of a midwife, the latter under the care of Dr. Carlstedt. The vertex presented at the superior strait, occiput posterior, uterus pretty firmly contracted around the child. The forceps were not used on account of the head being above the brim, and the tumid condition of the genitals. The hand was made to enter the uterus, and after some delay in pushing up the head, exploring the uterine cavity and turning the child, the delivery was completed. Drs. Carlstedt and Elliott assisted in the operation. The woman was left in good condition; the child was dead.

20. Mrs. S——, German, aged 40 years, had given birth to four children, two born dead and two living. The pelvis was contracted in its sacro-pubic diameter; she had been in charge of a midwife all day. The writer saw her at 6 p. m., May 14th, 1860. The waters had escaped three-fourths of an hour, funis in vagina, head at superior strait, but not engaged. The hand was introduced, the left foot secured and brought down, and the body brought away without difficulty; the head remained at the brim for half an hour, but was finally got away by aid of the vectis and blunt hook. The child was dead, but the woman was left in good condition.

21. Mrs. O. M——, aged 38, was taken in labor December 31, 1868; pains continued eighteen hours; the os uteri was well dilated, but the head did not engage in the superior strait; she had borne six children, all having been delivered by obstetrical operations, excepting, in one labor, when she brought forth twins, both being small. The pelvis was contracted and distorted, the sacral promontory being advanced toward the pubis, and inclining to the left side, thus dividing the entrance into the pelvis into two unequal halves. The membranes being still unbroken, and nothing contra-indicating, it was deemed advisable to proceed at once to delivery by turning. A still-born child was brought away in about half an hour; indeed, it appeared lifeless at an early period of the operation; the child was large, and the head passed the superior strait with much difficulty. The woman made a good recovery, and as will be seen further, was subsequently the subject of embryotomy.

22. Mrs. M——, aged 21, colored, who had borne one child, which she stated had been delivered with much difficulty and dead, was taken in labor Monday, September 24th, 1871. Professors Casselberry and VanNuys were in attendance ; strong pains continued uniil Tuesday, and then ceased; she had taken quite freely of ergot, probably enough to bring on its sedative effect on the uterus. On examination, the head was found low down, though still above the superior strait. The pelvis was distorted by the straits approximating together, and thus shortening the pelvic canal ; the sacral promontory was also advanced toward the symphysis pubis, being opposite the pubic arch ; the antero-posterior diameter measuring no more than two inches ; the child was dead and putrid. Forceps were deemed inadmissable, from the pelvic deformity ; the child was turned, and the feet brought down without difficulty ; the delivery of the body and head however was attended with some delay and trouble, their delivery occupying about an hour ; the blunt hook was used and the head perforated and reduced. The woman appeared to suffer no damage from the operation, but died however, eighteen hours after delivery. The cause of death is not known, but supposed to be from gangrene caused by injury from the protracted labor, possibly from simple exhaustion.

*Flooding.*—3 *Cases.*

23. Mrs. M——, about 25 years old, mother of three children, supposed to be eight months pregnant ; has been flooding to an alarming extent, followed by signs of prostration, for an hour ; no labor pains, os uteri dilatable, membranes whole ; version was decided upon, on account of the hemorrhage. The hand was passed into the uterus, and both feet secured and brought down. Both child and placenta came away without delay or trouble ; the child gasped for a short time, but finally expired, apparently from loss of blood. The hemorrhage ceased after delivery ; the uterus contracted promptly, and the woman made a good recovery.

24. Mrs. A——, aged about 29 years, has generally enjoyed fair health ; mother of three children ; has had several miscarriages, attended with troublesome flooding ; she was supposed to be at the full period of gestation. Hemorrhage commenced at 8 p. m., December 24th, 1861 ; a copious discharge, that resisted the remedies used, caused an examination to be made, which revealed a slightly open and rigid os uteri; the labor pains were apparently coming on ; at 10 o'clock the os was more dilated and dilatable ; flooding still profuse. It was not considered safe to delay the delivery longer, consequently the child was taken away by turning as soon as possible ; the patient, however, had sunk down and become pulseless, from loss of blood; the child was dead, exsanguine; the placenta, being free in the cavity of the uterus, was taken away. The patient was freely stimulated, and used opiates without avail ; the pulse was slightly restored about two hours after delivery, but from this time the woman continued to sink, and ceased to breathe at 4 p. m. of the 25th.

25. Mrs. R——, aged 25, has had only one child ; was injured by falling from a chair on which she was standing, at the end of seven months' gestation ; there was some laceration of the external labia near the symphysis pubis, excessive flooding, with symptoms of prostration, feeble labor pains, os moderately dilated, but rigid ; ergot and whiskey had been administered. The patient had been attended by Dr. E. T. Rennie. An effort to deliver by turning was agreed upon immediately after the writer had been called in, this being July 19th, 1870, whereupon the hand was passed through the membranes, after the patient had been brought under the influence of chloroform, the left foot brought down, and the child came forth without much trouble. The child was born dead ; the flooding ceased, and the woman made a good recovery.

*Eclampsia.—3 Cases.*

26. Mrs. N——, 20 years of age, primipara, eight and a half months pregnant, became delirious last night, this being March 6th, 1859, seeing stars, accompanied with other delusions of vision ; convulsions commenced about 8 o'clock this morning; the writer saw her about 9 o'clock, she having had three convulsions ; she does not recover her senses well between the paroxysms ; the pulse is strong, skin natural, condition comatose. About twenty ounces of blood having been taken from the arm, there was decided improvement in the circulation, with slight appearance of labor pains. On examination the os was found somewhat soft and dilatable ; delivery by turning was considered to be indicated. The hand was passed through the os uteri, slowly and not without difficulty, dilatation being effected by cautious manipulations with the fingers; finally the hand entered the uterine cavity, the feet were secured and brought down, and the delivery completed. The convulsions continued after delivery, causing apprehensions of a fatal result; they finally subsided, however, and the patient recovered; the child, too, gave every promise of doing well. The woman was quite insane for a month, but recovered. She is now the mother of several children, and has had no subsequent attack of convulsions.

27. Mrs. B——, 32 years of age, mother of one child, has had labor pains, more or less, for a month, sometimes strong and frequent. Yesterday, May 15th, 1872, she had three convulsions ; is of delicate and feeble constitution. On examination the os uteri was found moderately open and dilated. Dr. Casselberry had been in attendance, and he and the writer agreed that the labor should be promptly terminated by turning. After bringing the patient under the influence of chloroform, the hand was introduced through the cervix, meeting with considerable resistance from the rigidity of the os ; the feet were secured, and a small male child was brought away. After a lapse of about fifteen minutes the child commenced breathing, and appeared to be in good condition. The mother almost ceased to breathe during the delivery of the child, apparently from the effect of the anæsthetic ; placing her on the side, however, respiration was restored, and by the moderate administration of stimulants the pulse came up, so that an hour after delivery there was a fair promise of recovery. In the end the case resulted favorably for both mother and child.

*Forceps.*—13 *Cases. Inertia.*—9 *Cases.*

1. Mrs. F——, in labor, 1862, with the second child, the first having been delivered with forceps, has been suffering from chronic dysentery, with great tenderness over the abdomen ; has had labor pains all day ; child's head has remained in the same position at the inferior strait four hours ; the head presents in the first position. The forceps were applied and the delivery effected without accident. Mother and child both did well.

2. Mrs. F——, aged about 30, primipara, was taken in labor on the morning of January 19th, 1855, supposed to be at term; the head presented in the second position of the vertex, the membranes broke at 8 o'clock p. m. of same day; the head had descended to the floor of the pelvis by 10 o'clock, and remained stationary until 4 a. m. Dr. John T. Walker at this time was called in consultation, and delivery by forceps was agreed upon. The forceps being applied, the delivery was completed by 5 o'clock, the child coming away in good condition. The result was favorable for both mother and child.

3. Mrs. S——, aged 21 years, primipara, in labor February 17, 1859; labor pains had been strong for twelve hours; the child's head descended to the floor of the pelvis, and remained there without advancing, five hours; position of head, left occipit-acetabular. Further delay was deemed unsafe, the forceps were applied, and the child delivered; the child was alive, and both mother and child did well.

4. Mrs. D——, aged about 41, mother of two children; had been in labor under the care of a midwife all day; she was first seen by the writer at 10 o'clock p. m.; the pains had left her for two hours, after being good since 11 a. m. The head of the child was resting upon the brim of the pelvis, and had not entered the strait; occiput, left of pubis, in first position; the pelvis was small and the child's head large. The forceps were applied, and the child brought away without accident; child alive and strong, and the result all that could be desired.

5. Mrs. R——, aged 25, primipara, in labor December 19th, 1869; labor protracted, with symptoms of exhaustion; strong labor pains for eighteen hours; the head in the basin, above the floor of the pelvis, and ceased to descend; vertex presentation; the woman had been in good health; the child was large. Delivery by forceps was effected in fifteen minutes, under chloroform; there was slight central laceration of the perineum, which was measurably relieved by promoting granulation; the child was in good condition, and, upon the whole, the result was favorable for both mother and child.

6. Mrs. H——, aged 24, in labor eighteen hours, with first child, 12 m., March 29, 1871; had been in charge of another practitioner. The head was found in the basin, near the floor of the pelvis; no perineal tumor; the caput succedaneum was large and quite hard; the waters had been passed off four hours. After waiting an hour, and finding that the head did not advance sufficiently to give assurance that the child would be born alive by the natural powers, delivery by forceps was decided upon. The patient was already under the influence of the fluid extract of ergot, which had probably increased the force of the contractions, without advancing the labor. After securing partial insensibility by chloroform, the forceps were applied, and the delivery effected; the extraction of the head required the application of considerable tractile force; the head was in the second position of the vertex; the child was large; there was slight posterior laceration of the perineum; the child was alive, and the recovery of both mother and infant good.

7. Mrs. D——, aged 34, in labor March 6th, 1872; supposed her pregnancy had lasted ten months; has had labor pains off and on for two weeks; the os uteri was found open at 7 o'clock this morning; the labor continued with moderate pains until 4 o'clock p. m.; the vertex was at the lower strait, face in front, third position, with every indication of a large child. The forceps were applied, and the position changed by rotating the head from the third to the second position, and the child brought away, with but little difficulty or delay. The child weighed 11½ pounds; both mother and child did well.

8. Mrs. S——, aged about 20, primipara, taken in labor on the morning of August 29th, 1872; pains continued until daylight of 30th, then ceased entirely. Dr. Kennedy, the attending physician, had given a small portion of ergot, with some apparent effect. The head was found in the cavity of the pelvis; woman small; vulva rigid and contracted; the head above the floor of the pelvis: caput succedaneum very large. The forceps were adjusted without difficulty, and the head brought away be means of considerable tractile effort. The perineum was lacerated to, but not through, the sphincter ani, otherwise nothing untoward as regards the mother resulted; the child, however, being a large boy, was asphyxiated, but finally had respiration established; unfortunately, however, at the expiration of an hour, it ceased to breathe.

9. Mrs. B——, aged 26, primipara, healthy excepting undue nervousness from domestic trouble; was taken in labor about midnight of February 11th, 1876; os tincæ open about 6 p. m.; the head of the child descended to the floor of the pelvis by 8 a. m., and remained in this position, with no perceptible change, until 11 a. m.; face of the child in front and toward the right acetabulum (fourth position of vertex presentation). The child was brought away by moderate traction by the forceps; slight laceration of perineum; child large, but weight not ascertained; asphyxiated for a few minutes. Finally, both mother and child made a good recovery.

*Contracted Pelvis.*—2 *Cases.*

10. Mrs. B——, aged 20, greatly deformed pelvis, distorted and contracted at lower outlet; taken in labor this Sunday morning, July 1st, 1860, the pains continuing all day; the waters escaped at 7 o'clock p. m., and the head descended to the floor of the pelvis by 8 o'clock. The head presented, with face anterior and to the left. The woman's strength had been reduced by frequent vomitings, during the last three months; the child's head remaining stationary, and the woman's health declining rapidly, it was deemed advisable to terminate the labor by the aid of forceps; this was accomplished without difficulty; the child was a large boy. On examination, the recto-vaginal septum was found to be lacerated low down; this injury could not be traced as having resulted from the use of forceps, as the instrument did not appear to press unduly upon the part injured; the passage of the large head of the child through the contracted lower strait, the occiput being posterior, probably gave rise to the accident. The mother and child were both saved.

11. Mrs. O'M—— is the patient referred to in case 21 of version cases. Her present labor commenced February 3d, 1855; she had previously borne two children, after difficult labors, both being still-born; pelvis contracted and distorted at upper strait, the brim resembling the figure 8, with the largest opening at the left. The waters passed away, but the head remained at the superior strait; after waiting eight hours, the head still remaining stationary, with pains abating and the patient's vital powers on the wane, the forceps were resorted to; after some delay, and the application of strong tractions, the child came away; it was still-born, and weighed 12 pounds. The woman recovered without further accident.

*Eclampsia.*—2 *Cases.*

12. Mrs. O——, primipara, aged 20, in labor September 5th, 1855; seized with convulsions about 11 o'clock a. m.; head of child low in excavation; presentation and pains favorable. The head was delivered by the aid of forceps, the operation occupying fifteen minutes; laceration of perineum three-fourths of an inch, through which a stitch was applied. The patient had one fit after delivery, but both mother and child did well in the end.

13. Mrs. W——, 19 years of age, first child; had been in labor five hours, February 22d, 1868. Dr. E. T. Runcie was in attendance, and assisted in the delivery; the patient had two convulsions, with an interval of fifteen minutes, the last, one hour and a half ago; coma continued for nearly an hour after the fit; child's head resting on perineum; pains good; first position of vertex presentation. Delivery by forceps was effected, without trouble or loss of time; result favorable for both mother and child.

*Cephalotomy.*—2 *Cases.*

1. Mrs. M——, aged 34, primipara; pelvis contracted at superior strait; waters have passed off thirty-six hours; child apparently dead; head presenting at brim, where it has remained fixed for twenty-four hours; pains pretty good; the child was turned, and the body brought away without difficulty; the head failing to follow, the cranium was perforated posterior to the foramen magnum, the brain was scooped out, and by aid of the crotchet the head was taken away. The woman was uninjured, and made a good recovery. Date, October 19th, 1860.

2. Mrs. O'M——, about 42 years of age, has had several children, all delivered by artificial aid; pelvis contracted by promontory of sacrum advancing toward symphysis pubis, and inclining to right side; was taken in labor September 29th, 1864, and was under the care of Dr. DeBruler. Dr. John T. Walker, and afterwards the writer, were called in consultation. The waters had been discharged about eight hours, the funis protruded externally and was devoid of pulsation, and had been so for several hours; the head was resting upon the pelvic brim. No care being necessary in regard to the child, it being dead, it was decided that the mother's welfare would be best subserved by immediate delivery; whereupon, the head was reduced by cephalotomy, and after an hour's exertion the child was brought away. In this case, after the head came down, the body was made to enter the upper strait with some difficulty; the child passed down on the left side of the strait, this side being less contracted than the right. Smallie's scissors, the crotchet and the blunt hook were the instruments used for effecting the delivery. The woman made a good recovery.

### *Remarks on Obstetrical Operations.*

Version has generally been resorted to in vertex cases, when the head was still above the brim and immediate delivery became necessary, from the belief that it was safer for the woman than the forceps, that it could be practiced when an attempt at forceps delivery might fail, and that even to the child the danger from delivery by turning would be no greater than by the forceps when applied above the strait; and finally, because the operation in many instances would be more expeditious. Even after the head has descended into the excavation, and is still easily pushed up above the brim, turning has sometimes been preferred, especially where the child was dead. Turning, as compared to cephalotomy, has been regarded as safer for the mother, the cephalotomy being a dernier resort, and to be used only when turning is impracticable. In bringing down the feet, securing a single foot has been deemed sufficient, if for no other reason to save the valuable time necessarily consumed in searching for the second foot, and also from the belief that by leaving the second limb folded against the abdomen, some protection is afforded the funis against undue pressure. Ample experience has convinced the writer that the delivery can be effected as well by securing one foot as two.

In placenta prævia cases, the indication has been supposed to be two-fold: 1, to arrest hemorrhage, that threatens the woman's life; and, 2, to save the child's life. To secure these advantages, delivery by turning as soon as it can be done, without using too much violence in the introduction of the hand, is the chief, if not the only resource. Therefore, at the earliest practicable moment, the hand is to be passed through the cervix into the cavity of the uterus, detaching the placenta from its connection as little as possible, and when the membranes are reached, the fingers are to be pushed through them, search made for the child's feet, or rather foot, which being brought into the vagina, the child is to be cautiously taken away. The operation need not be delayed on account of the absence of labor pains, nor of the immature state of the gestation. To wait in these cases for the natural process of labor, would result too frequently in the double loss of mother and child.

In craniotomy, after the brain has been removed and the cranium collapsed, it is frequently still best to resort to turning, and bringing the child away by the feet, this being safer for the woman than drawing it away head first, by means of tractions by the crotchet. In flooding cases, other than those of placenta prævia, turning by the feet will generally be found the most expeditious method of delivery, excepting, however, in those cases wherein the head has already been pushed into the pelvic basin by uterine contractions. Podalic version has been resorted to in puerperal convulsions, at as early a period as will admit of the introduction of the hand, not with the view that delivery will always cure the convulsions, but that evacuating the uterus removes a potent cause of irritation, and places the woman in a condition much more favorable to recovery.

As before stated, the forceps have not often been applied above the brim, this mode of using them, indeed, appearing more intended to show what may be done than for any special advantage or indication. After the head has passed the superior strait, as a general rule, the forceps will be the best and safest remedy; and, should the head have been pushed into the excavation by strong pains, and yet its expulsion not promised by the efforts of nature, the forceps would be indicated, and turning should not be thought of. The writer has never applied the instrument to any other part of the child than the head; the barbarous practice of applying them to the pelvis, should the child be delivered alive, might so crush the bones as to endanger permanent damage to the pelvis. The forceps, then, being applied to the head only, should be as nearly as practicable adjusted to its sides. The writer has never met with a case in which application over the face and occiput was necessary; when convenient, the blades have been adjusted to the sides of the woman's pelvis, but this has been deemed less important than to permit much obliquity in their application, as regards the child's head. Indeed, in oblique positions of the head, which constitute more than 90 out of 100 of the cases, it is impossible to apply the instrument squarely to both pelvis and head. When practicable, the forceps have been removed before the escape of the head from the vulva, to avoid laceration; but this practice is more easily recommended than followed, inasmuch as the moment the head has engaged fairly in the outlet, it will frequently escape through the vulva, even before the operator finds himself able to remove the instrument. Doubtless lacerations are more frequent than they otherwise would be, in consequence of this failure, but in most cases requiring the use of the forceps lacerations would be liable to follow a natural delivery.

Cephalotomy has been recommended by the writer, only in those cases in which neither turning nor forceps could be made available. It is not regarded as a safe operation for the woman, and when a fatal result is avoided she is necessarily exposed to more or less danger of laceration about the cervix and vagina, so that after the bulk of the child's head has been reduced by the evacuation of the brain, the delivery may be completed more safely and more expeditiously by podalic version than by tractions with sharp and powerful instruments. In addition to this, we may urge the risk of bruising and tearing the soft parts by drawing away fragments of the fœtal skull, that are almost certain to become loosened during the transit of the head.

When the cephalotribe is required in extreme contraction of the straits, a resort to turning will generally be inadmissible, from the difficulty or impossibility of drawing a full-sized child through the pelvic canal. When, indeed, it is found necessary to use this instrument to crush the head, its power will generally be required also to reduce the other bulky parts of the child.

